# Study of HOXB13 Gene Variants in Prostate Cancer Patients

**DOI:** 10.7759/cureus.72513

**Published:** 2024-10-27

**Authors:** Kazhal M Sulaiman, Rebwar M Hama Salih

**Affiliations:** 1 Department of Biology, College of Education, Salahaddin University-Erbil, Erbil, IRQ

**Keywords:** hoxb13, mutations, prostate cancer, prostate-specific antigen, risk factors

## Abstract

Background: Prostate cancer (PC) is a prevalent malignancy with a significant hereditary component. The HOXB13 gene, encoding a transcription factor involved in prostate development, has been implicated in PC risk.

Objective: The objective of this study was to assess the existence of HOXB13 mutations in PC patients.

Method: The retrospective study included 33 PC patients and 23 controls. Demographic data, family history, and smoking habits were recorded. Prostate-specific antigen (PSA) levels were measured. We investigated the second exon of HOXB13 after extracting genomic DNA from blood samples for mutations using polymerase chain reaction and Sanger sequencing.

Result: PC patients had a higher mean age (64.7 years), more frequent positive family history (63.64%, N = 21), and higher smoking prevalence (60.61%, N = 20) compared to controls. PSA levels were significantly elevated in patients (76.58 ng/ml) versus controls (7.22 ng/ml). HOXB13 mutations, including thymine (3.03%, N = 1), guanine (27.27%, N = 9), and adenine (33.33%, N = 11) mutations, were observed in patients, while no mutations were found in controls.

Conclusion: PC patients had higher mean age, more positive family histories, higher smoking rates, and elevated PSA levels. HOXB13 mutations were significantly higher in patients compared to controls. These findings emphasize the roles of HOXB13, age, family history, smoking, and PSA in PC risk stratification.

## Introduction

Prostate cancer (PC) poses a significant global health burden, being the sixth most common cause of cancer-related mortality of all the most common malignancies among males, following lung cancer [1]. Benign prostatic hyperplasia (BPH) is associated with a favorable 100% five-year survival probability [2].

Familial history significantly increases PC risk, affected by the number of afflicted relatives in the immediate family and early-onset cancers in the family, particularly diagnoses at or before age 55 [3]. Despite the recognized familial component, identifying highly penetrant genes in hereditary PC has proven challenging. Nonetheless, identifying individuals with germline mutations at higher risk for PC is becoming increasingly important [4].

The management of PC is multifaceted, with treatment options for localized tumors including radiation therapy and radical prostatectomy, while antiandrogen therapy is used for metastatic types [5]. However, a significant challenge arises in the form of castration-resistant PC, wherein 20-30% range, cancers can acquire resistance to hormonal therapy, leading to increased aggressiveness [6].

Development of sex organs, cell proliferation, and embryonic axial differentiation are all regulated by transcription factors encoded by the HOX gene [7]. HOXB13, a member of the HOX gene superfamily, has garnered significant attention due to its extensive pattern of expression, from low levels in the rectum and colon to high levels in a healthy prostate [8].

HOXB13 is essential for prostate development and cancer. Multiple HOXB13 mutations are linked to a higher risk of developing PC [2,9,10].

There are genetic variations across different populations considering PC risk. The G84E mutation is the most well-known mutation associated with PC, particularly prevalent in patients of European ancestry [10]. Other germline mutations of HOXB13, such as G135E in Chinese populations [11] and p.(Ala128Asp) and p.(Phe240Leu) in Portuguese populations [12], also correlate with increased PC risk. Additionally, research revealed that the HOXB13 mutation occurs more frequently in people with a family history of PC and those who develop the disease at an early age, compared to those who are healthy [13-17].

Therefore, our research intended to assess the existence of HOXB13 mutations in PC individuals.

## Materials and methods

This retrospective study involved 56 specimens overall, comprising 33 cases of PC (patient group) and control cases (control group), obtained from a urology-nephrology facility in Erbil (Nanakali Hospital for Blood Diseases and Cancer) between March 2023 and July 2023. During the study period, most of the subjects who were old had BPH and did not have the characteristics of a healthy person as a control person. As a result, 23 people were selected as healthy individuals who did not have any type of prostatic hyperplasia (BPH). Written informed consent was obtained from all participants before they could be included in the investigation. Clinicopathologic information, including age, family history of PC, and smoking habits, was gathered and documented for each participant. Venous blood samples were collected from PC patients and stored at -80°C in ethylenediaminetetraacetic acid (EDTA)-containing tubes to facilitate DNA extraction. The Ethical Committee of Salahaddin University-Erbil, Iraq approved the study protocol. The prostate-specific antigen (PSA) test was conducted following the methodology described by Barry [18], utilizing the Elecsys total PSA kit (Roche Diagnostics, Risch-Rotkreuz, Switzerland) according to the manufacturer's manual. The procedure involves collecting a 5-10 mL blood sample from the patient’s arm. To ensure accuracy, patients were instructed to abstain from sexual activity for 24 hours prior to the test and to inform their healthcare provider about any medications they were taking. Generally, elevated PSA levels are indicative of an increased likelihood of PC.

Extraction of genomic DNA from PC tissues and blood samples of patients using the genomic DNA extraction kit (Add Bio, Daejeon, Korea) was done following the manufacturer's instructions. Briefly, the blood sample was treated with binding buffer and proteinase K at 60°C for 15 minutes. After adding ethanol, the mixture was transferred to a column, washed twice, and the DNA was then precipitated. The quality of the extracted DNA was assessed by gel electrophoresis, while its quantity was determined using a NanoDrop spectrophotometer (Thermo Fisher Scientific, Waltham, MA).

The second exon of the HOXB13 gene (NM_006361.5) was analyzed for genetic variants using polymerase chain reaction (PCR). The primer sequences utilized were as follows: forward primer: 5'GCCTGTAGGGTGACCTGTGT'3; reverse primer: 5'TCCGTCTCCCTGCACATACT'3. The PCR reaction mixture totaled 25 μL, comprising 2.4 μL of 2× reaction buffer, 2 μL of genomic DNA, 0.1 μL of Taq DNA polymerase (5 U/μL), 1 μL of each primer, 1.5 μL of magnesium chloride (50.0 mM), and 0.5 μM of deoxynucleotide triphosphate (10.0 mM). Initially, the samples were denatured at 94°C for three minutes. Using an AlphaMax thermocycler (PHARMAQ, Overhalla, Norway), 35 cycles were performed for exon 2, each involving a 30-second denaturation at 94°C, a 30-second annealing at 62°C, a one-minute extension at 72°C, and concluding with a 10-minute final extension at 72°C.

The amplified PCR products were run on a 1.5% agarose gel and stained with a solution of 3.5 μg/mL green viewer to verify the expected fragment sizes. Macrogen, Inc. in Seoul, South Korea then conducted direct sequencing of the PCR products using the traditional Sanger technique. The genome sequencing data were analyzed using the Mutation Surveyor program from SoftGenetics, based in State College, PA.

Statistical analysis

Data analysis was conducted with SPSS version 27 (IBM Corp., Armonk, NY). Normality was evaluated using the Shapiro-Wilk test and histograms. Quantitative parametric data were reported as mean and standard deviation and analyzed via an unpaired Student's t-test. Quantitative non-parametric data were presented as median and interquartile range (IQR) and analyzed using the Mann-Whitney test. Qualitative data were expressed as frequency and percentage and analyzed with the chi-square test or Fisher's exact test when applicable. A two-tailed p-value < 0.05 was deemed statistically significant.

## Results

The demographic data revealed significant differences between the patient and control groups. The patient group had a higher mean age of 64.7 ± 9.8 years compared to 58.83 ± 11.76 years in the control group (p = 0.047). Additionally, a family history of PC was more prevalent in the patient group, with 63.64% (N = 21) reporting a positive family history, as opposed to only 26.09% (N = 6) in the control group (p = 0.006). The smoking habit was also significantly higher in the patient group, with 60.61% (N = 20) of patients being smokers, compared to only 13.04% (N = 3) in the control group (p < 0.001) (Table 1).

**Table 1:** Demographic data of the studied groups. Data are presented as mean ± SD or frequency (%). * Significantly different as p-value ≤ 0.05.

	Patient group (n = 33)	Control group (n = 23)	P-value
Age (years)	64.7 ± 9.8	58.83 ± 11.76	0.047*
Family history	21 (63.64%)	6 (26.09%)	0.006*
Smoking habit	20 (60.61%)	3 (13.04%)	<0.001*

The mean PSA level in the patient group was 76.58 ± 27.42 ng/ml, which was substantially higher than the mean level of 7.22 ± 4.23 ng/ml observed in the control group (p < 0.001) (Table 2).

**Table 2:** Prostate-specific antigen (PSA) level of the studied groups. Data are presented as mean ± SD. * Significantly different as p-value ≤ 0.05.

	Patient group (n = 33)	Control group (n = 23)	P-value
PSA (ng/ml)	76.58 ± 27.42	7.22 ± 4.23	<0.001*

The distribution of HOXB13 gene mutations differed significantly between the patient and control groups (p < 0.001). In the patient group, 3.03% (N = 1) had a thymine mutation, 27.27% (N = 9) had a guanine mutation, 33.33% (N = 11) had an adenine mutation, and 24.24% (N = 8) had a sequence error. In contrast, none of the individuals in the control group exhibited any HOXB13 gene mutations (Table 3).

**Table 3:** Type of mutation of the studied groups. Data are presented as frequency (%). * Significantly different as p-value ≤ 0.05.

	Patient group (n = 33)	Control group (n = 23)	P-value
Thymine mutation	1 (3.03%)	0 (0%)	<0.001*
Guanine mutation	9 (27.27%)	0 (0%)	
Adenine mutation	11 (33.33%)	0 (0%)	
Sequence error	8 (24.24%)	0 (0%)	
No mutation	4 (12.12%)	23 (100%)	

## Discussion

PC is a major global health issue. Understanding its demographic, clinical, and genetic factors aids in better risk stratification, early detection, and personalized treatments [4,7,13,19]. Identifying specific genetic mutations is crucial for understanding the disease, assessing risks, and developing targeted therapies [2,8,20,21].

Our study revealed significant demographic differences between the patient and control groups. The PC patient group had a higher mean age of 64.7 ± 9.8 years compared to 58.83 ± 11.76 years in the control group (p = 0.047). This finding is consistent with the well-established association between advanced age and increased PC risk, as highlighted by various studies [4,7,13,19]. The increased incidence of PC with age can be attributed to cumulative exposure to environmental and genetic risk factors over time, as well as age-related changes in hormonal levels and cellular processes [13].

Furthermore, our study found a significantly higher prevalence of a positive family history of PC in the patient group (63.64%) compared to the control group (26.09%) (p = 0.006). This observation aligns with the findings of several other studies, which have consistently identified a significant risk factor for PC is having a family history of the disease [4,7,13]. The guidelines from the National Comprehensive Cancer Network advise genetic testing for individuals with a notable family history of PC or associated malignancies [7]. A history of PC in the family might suggest the existence of inherited genetic variants or shared environmental exposures that contribute to the development of PC [13].

Interestingly, our study also revealed a significantly higher smoking habit in the PC patient group (60.61%) compared to the control group (13.04%) (p < 0.001). This finding is supported by previous meta-analyses and cohort studies that have demonstrated a modest but statistically significant increased risk of PC incidence and mortality among smokers [1,22-24]. The potential mechanisms underlying this association may involve hormonal or genetic factors, such as altered metabolism of polycyclic aromatic hydrocarbons present in cigarette smoke [1,24].

Our study found significantly elevated PSA levels in the PC patient group (76.58 ± 27.42 ng/ml) compared to the control group (7.22 ± 4.23 ng/ml) (p < 0.001). In comparison to other studies, our observed PSA levels in the patient group appear to be higher than those reported in the literature. This observation is consistent with the well-established role of PSA as a biomarker for PC detection and monitoring as reported by Boyle et al. [19], who reported a median PSA level of 16.1 ng/mL (IQR: 6.8-87.0 ng/mL) at diagnosis in their cohort of metastatic PC patients. Similarly, Elsesy et al. [6] found a median PSA value of 8 ng/mL (range: 2.1-165 ng/mL) in their patient cohort, with a majority (60.2%) having PSA levels below 10 ng/mL. The higher PSA levels observed in our study could be attributed to factors such as disease stage, tumor burden, or the specific patient population under investigation.

Interestingly, the studies by Li et al. [23] and Rawla [1] explored the potential influence of smoking on PSA levels and PC risk. While Li et al. [23] found that current and former smokers had significantly lower total PSA and percent free PSA levels compared to never smokers, Rawla [1] indicated a potential link between smoking and elevated mortality rates in PC and aggressive disease, despite not conclusively linking it to increased incidence. These findings highlight the complex interplay between lifestyle factors, biomarkers, and PC outcomes, warranting further investigation.

Our study revealed a significant difference in the distribution of HOXB13 gene mutations between the PC patient and control groups (p < 0.001). In the patient group, 3.03% had a thymine mutation, 27.27% had a guanine mutation, 33.33% had an adenine mutation, and 24.24% had an unspecified error, while none of the individuals in the control group exhibited any HOXB13 gene mutations. These results align with the widely recognized function of the HOXB13 gene as a predisposing factor for PC (Appendices) [2,4,8,20].

The HOXB13 gene produces a regulatory protein crucial for diverse cellular functions such as cell growth, specialization, and proliferation [8]. Changes in the HOXB13 gene, notably the G84E mutation, have been linked to a heightened susceptibility to PC, particularly among those with a family history of the condition [4,20]. Roudi et al. [2] identified several variants in the HOXB13 gene, including c.366C>T, c.513T>C, and a novel variant c.127A>G in exon 2, which may influence the risk of PC.

It is worth noting that the study by Marlin et al. [21] discovered a rare heterozygous germline variant c.853delT (p.Ter285Lysfs) rs77179853 in the HOXB13 gene among African ancestry PC patients, with a minor allele frequency of 3.2%. This variant correlated with a history of prostate and breast cancers within families, highlighting the potential role of HOXB13 mutations in hereditary PC risk across different populations.

Furthermore, Patel et al. [8] demonstrated that while HOXB13 protein expression was observed in localized PC, lower expression levels were associated with higher-grade tumors. Additionally, advanced metastatic PC, particularly neuroendocrine PC, exhibited reduced HOXB13 expression due to increased gene body CpG methylation. These findings suggest that HOXB13 alterations could potentially influence the onset and advancement of PC, with potential implications for disease management and targeted therapies.

However, the following are the limitations of the study. This study is a retrospective analysis, not a case-control study, which is important to clarify in discussing the methodology. Retrospective studies rely on previously collected data, limiting the ability to manipulate the sample size or adjust sampling procedures. The sample population used for this research was determined by the availability of participants, and thus the control group was selected based on the characteristics of the available healthy individuals. Specifically, many of the older individuals in the sample who presented at the hospital had BPH, making it difficult to find a large number of healthy, older men without prostate issues for the control group. Consequently, the control group included individuals with a slightly broader age range to ensure an adequate number of healthy participants. Without this adjustment, it would have been challenging to create a control group with enough healthy subjects for valid comparisons.

## Conclusions

PC patients had higher mean age, more frequent positive family history, and higher smoking prevalence. Elevated PSA levels were observed in patients. HOXB13 mutations, including thymine, guanine, and adenine mutations, were significantly higher in patients compared to controls with no mutations. These findings highlight the role of HOXB13, age, family history, smoking, and PSA in PC risk stratification. Considering the direct and positive role of HOXB13 mutations in the development of PC, identification of HOXB13 mutations in the screening program can be given more attention, and with early detection, appropriate treatment strategies and measures can be adopted. Therefore, this study underscores the role of the HOXB13 gene in PC, emphasizing the higher prevalence of mutations in PC patients compared to controls. While the study's sample size and retrospective nature impose limitations, including the broader age range of the control group, the findings provide valuable insights into genetic predisposition in PC. The genetic component remains a key factor in understanding PC risk, and further studies with larger and more diverse populations are needed to confirm these findings and explore additional genetic markers. Early identification of genetic mutations such as those in HOXB13 may improve risk stratification and potentially guide more personalized treatment approaches. A larger sample size and study in different medical centers and also the investigation of other genetic factors involved in the development of cancer are necessary for future studies.

## Figures and Tables

**Figure 1:**
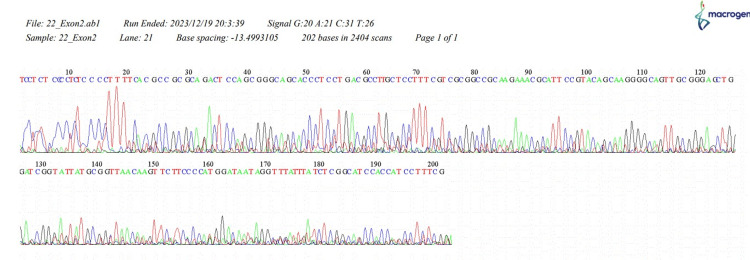
The sequence of HOXB13, 22_Exon2.

**Figure 2:**
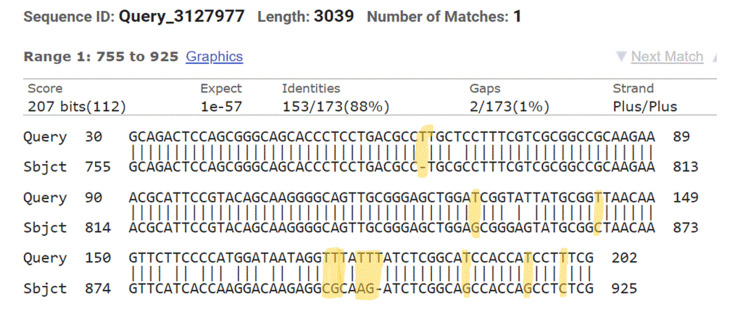
NCBI BLAST. Query is our results, while subject is the sequence in NCBI. Yellow marks are the identified mutation. NCBI: National Center for Biotechnology Information; BLAST: Basic Local Alignment Search Tool.

**Figure 3:**
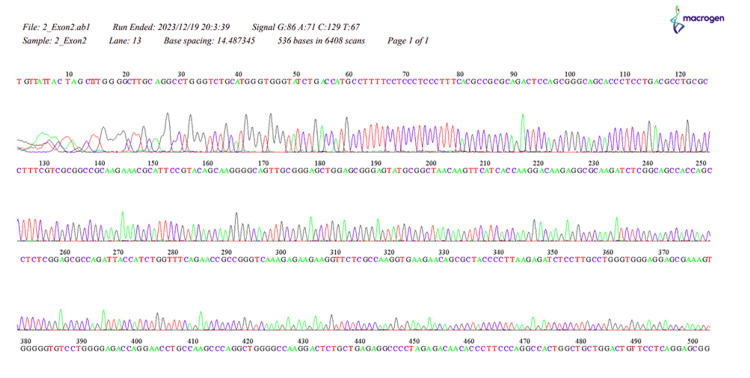
The sequence of HOXB13, 2_Exon2.

**Figure 4:**
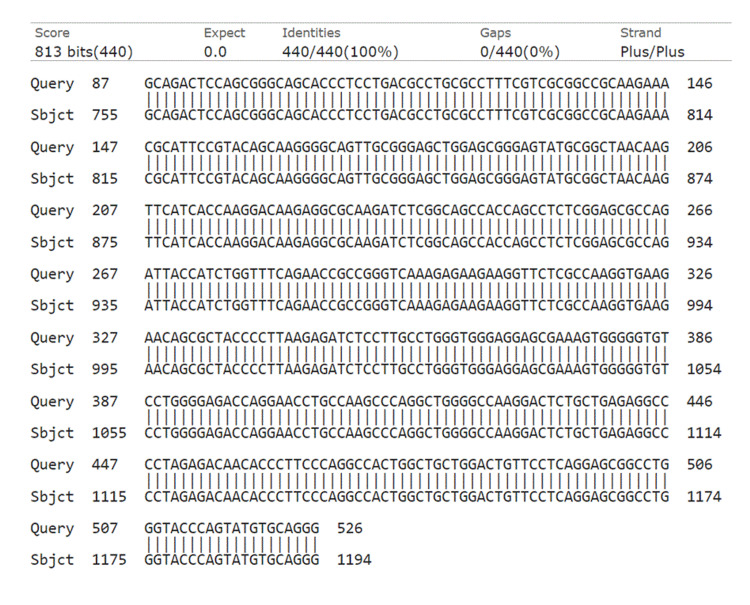
NCBI BLAST. Query is our results, while subject is the sequence in NCBI. No mutations are identified. NCBI: National Center for Biotechnology Information; BLAST: Basic Local Alignment Search Tool.

**Figure 5:**
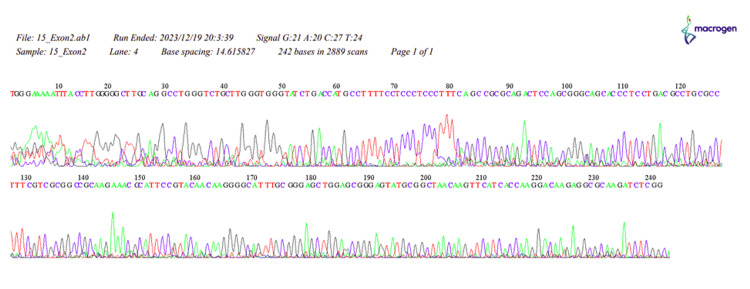
The sequence of HOXB13, 15_Exon2.

**Figure 6:**
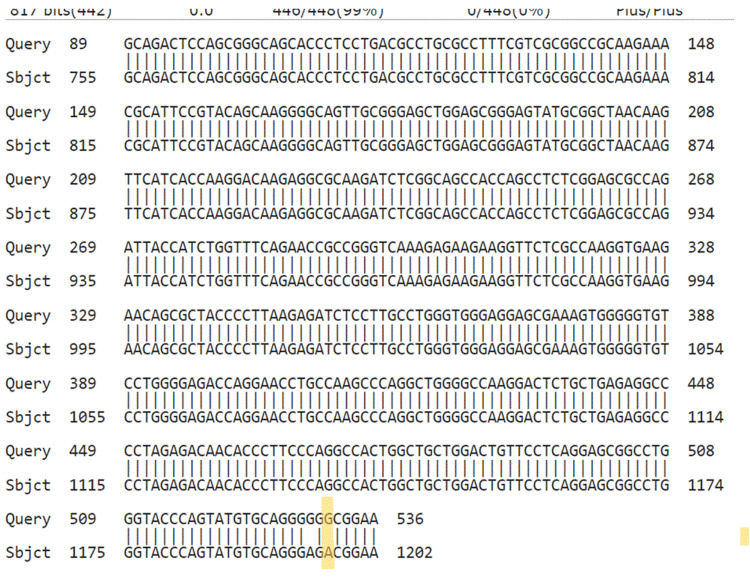
NCBI BLAST. Query is our results, while subject is the sequence in NCBI. Yellow marks are the identified mutation. NCBI: National Center for Biotechnology Information; BLAST: Basic Local Alignment Search Tool.

**Figure 7:**
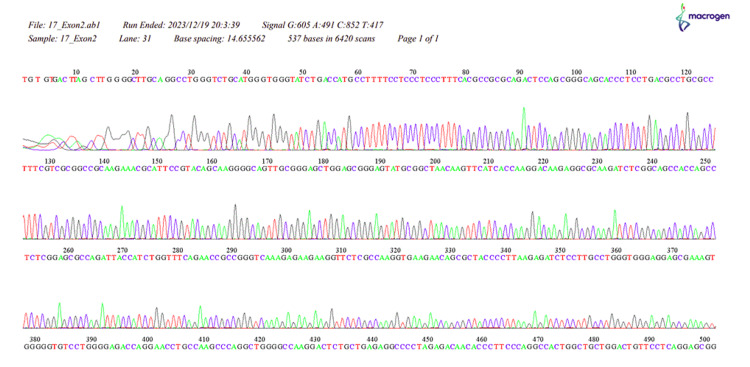
The sequence of HOXB13, 17_Exon2.

**Figure 8:**
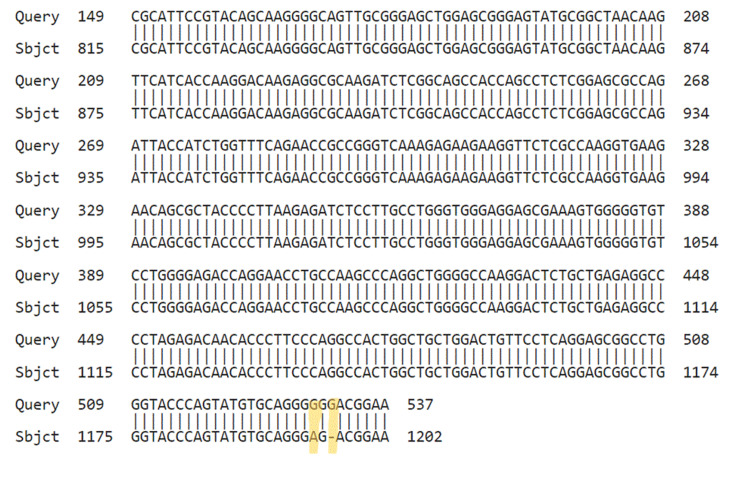
NCBI BLAST. Query is our results, while subject is the sequence in NCBI. Yellow marks are the identified mutation. NCBI: National Center for Biotechnology Information; BLAST: Basic Local Alignment Search Tool.

**Figure 9:**
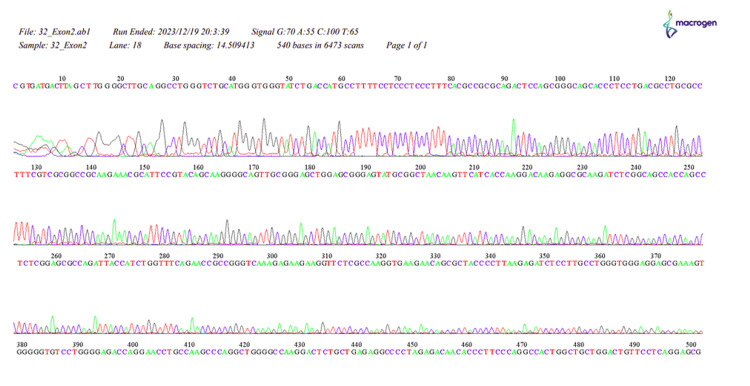
The sequence of HOXB13, 32_Exon2.

**Figure 10:**
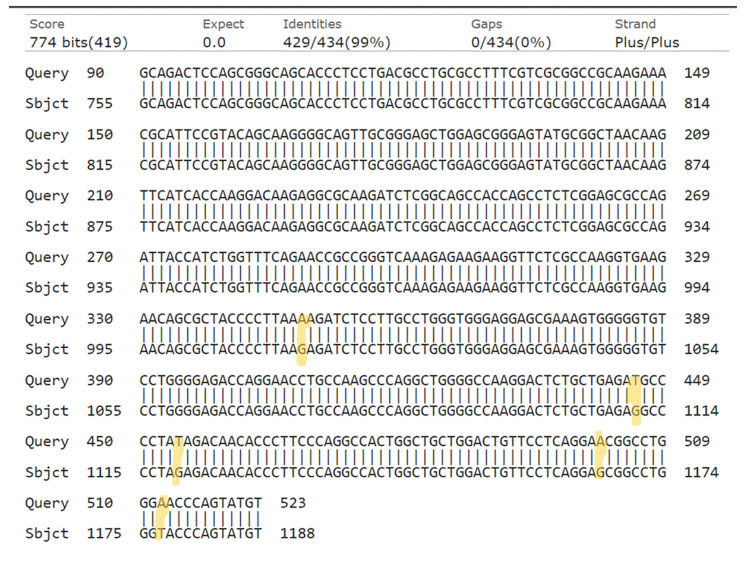
NCBI BLAST. Query is our results, while subject is the sequence in NCBI. Yellow marks are the identified mutation. NCBI: National Center for Biotechnology Information; BLAST: Basic Local Alignment Search Tool.
